# Fermentation Efficiency of Rice Bran, Rice Polish, and De‐Oiled Rice Bran for Nutritional Upgrading and Bioethanol Production

**DOI:** 10.1002/fsn3.71382

**Published:** 2025-12-26

**Authors:** Syful Islam, Md. Abdullah Bijoy, Md. Aliar Rahman, Rakhi Chowdhury, Khan Md. Shaiful Islam

**Affiliations:** ^1^ Department of Animal Nutrition Bangladesh Agricultural University Mymensingh Bangladesh

**Keywords:** bio‐ethanol, de‐oiled rice bran, fermentation, nutrition, rice bran, rice polish

## Abstract

Rice Bran (RB) and Rice Polish (RP) are by‐products of the rice milling industry where further oil extraction from RB produces De‐oiled Rice Bran (DORB). These by‐products are often underused because they contain anti‐nutritional factors (ANFs), have a high fiber content, and limited nutrient availability. This study aimed to improve the nutritional potentiality through yeast fermentation. RB, RP and DORB were employed in a 2 × 3 × 9 factorial experiment with Baker's yeast (*Saccharomyces cerevisiae*) at 1.0% and 2.0% level, with three moisture levels (40%, 50%, 60%) and 9 times (0, 6, 12, 18, 24, 30, 36, 42, 48 h), incubated at 39°C in 100 mL glass syringe. The fermentation efficiency was measured by cumulative gas production, ethanol yield, change in proximate composition, and availability of phosphorus (AvP). Fermentation of RB was maximum, with 2932 mL/100 g DM gas at 6 h and 3.49 g/100 mL ethanol at 48 h at 60% moisture level and 2.0% yeast. Its crude protein (CP) was raised by 23.1%, crude fiber (CF) was lowered by 20.1%, ether extract (EE) doubled nearly, metabolizable energy (ME) by 27.0%, and AvP. 0.27–0.42. RP was found as moderately fermentable by 713 mL/100 g DM gas and 1.45 g/100 mL ethanol, whereas nutritional improvement was noticed with an 18.2% reduction of CF, a 66% increase in EE, a 9.5% increase in ME, and AvP from 0.95 to 1.26. DORB was least fermentable but showed considerable nutritional improvements, including a 15.6% improvement in CP, an 18.9% reduction in CF, a 294% increase in EE, a 22% increase in ME, and a change in AvP from 0.82% to 1.02%. Fermentation significantly improved the nutritional potential of RB, RP, and DORB, with RB having the highest protein, ME, and AvP. Though RB was most fermentable, RP was partially improved, whereas DORB, despite low fermentability, had high protein, fiber removal, and AvP.

## Graphical abstract

Yeast fermentation significantly improved the nutritional value of rice bran (RB), rice polish (RP), and de‐oiled rice bran (DORB), with RB showing the highest gas and ethanol production along with major increases in crude protein, metabolizable energy, and available phosphorus. RP exhibited moderate but stable fermentation, while DORB showed limited fermentability yet substantial nutritional upgrading. Overall, the optimal fermentation condition: 2%, 60%, and 30–36 h; produced the greatest enhancements across all substrates.

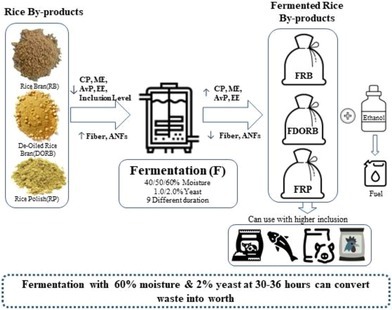

## 1 Introduction

Rice is a staple food for almost one‐fourth of the human race (Gul et al. 2015) with the global production rising to 540.93 MMT in 2024/25 with the further contribution of 36.6 MMT from Bangladesh (USDA 2025). Rice milling industries produce large amounts of agro‐industrial by‐products like rice bran (RB), rice polish (RP), and de‐oiled rice bran (DORB). These by‐products can be used in animal feed to reduce production costs and to ensure sustainability (Shah et al. 2025) along with bioconversion and eco‐friendly recycling processes (Chatterjee and Tiwari 2025). Where Clark and Tilman (2017) reported that using agricultural waste and by‐products in feed can decrease 20% environmental impact of livestock production.

Among these by‐products, RB contributes approximately 8.0%–10.0% of the total rice (Heuzé and Tran 2015), which corresponds to a global production of approximately 54.09 MMT in 2024/25. RB is an economically valuable by‐product because it contains a high oil (12%–22%), protein (11%–17%), and fiber (6%–14%) with minor micronutrients like tocopherols, oryzanol, and sterols (Devi et al. 2021). For these benefits, RB is largely used in poultry, fish, and cattle feed (Ranjan et al. 2022). However, its nutritional disadvantages (high fiber level) and the occurrence of anti‐nutritional factors (ANFs) such as phytic acid (Shuvo et al. 2022), which limit its digestibility and mineral availability in monogastric animals (Chuang et al. 2021).

Rice polish, valued at 3.0% of rice output (Heuzé and Tran 2015), is a significant source of feed ingredient. In 2024/25, its global outputs totaled approximately 16.23 MMT. RP is characterized by well‐proportioned nutrient contents like protein (13.2%–17.1%), fat (14.0%–22.9%), fiber (9.5%–13.2%), vitamins and antioxidants like γ‐oryzanol (Hossain et al. 2012). In addition, it has superior amino acid profiles compared to wheat or maize (Khalique et al. 2004). Using RP as an energy source in cattle feed can increase milk yield (Flores‐Cocas et al. 2021), where RP sugar cane silage diets can increase nitrogen retention, digestibility and growth performance in sheep (Vega Granados et al. 2019). However, similar to RB, it has ANFs (phytates) that cause depression of feed intake and production performance (Khalique et al. 2003).

De‐oiled rice bran is derived after the removal of oil from RB and is widely used as a cheap feed supplement (Kumar et al. 2019; Pal and Pratap 2017). Its worldwide economic importance has been estimated at USD 957.7 million in 2024 and is expected to grow to USD 1362 million by 2032 (Data Bridge Market Research 2025). DORB contains about 14.0% crude protein, 14.9% fiber, 11.2% ash, and 59.1% nitrogen‐free extract on a nutritional basis (Ranjan, Sahu, Deo, and Kumar 2018). A previous study revealed that DORB at 60% inclusion in *L. rohita* fingerlings' body organs responded to higher oxidative stress (Kumar et al. 2018). While possessing anti‐oxidative activity via oryzanol (Devi and Arumughan 2007), DORB is restricted in its use via ANFs such as oxalates, saponins, phytates, and tannins (Sharma et al. 2018) and can easily be replaced by potato leaf meal without any significant effect in *L. rohita* diet (Zishan Ahmad et al. 2019). Where fermentation increases fatty acid content with reduction of phytate and trypsin inhibitor (Ranjan et al. 2019).

Rice producing countries had great advantages of using these valuable by‐products at cheap cost (Kumar et al. 2019) but the key problems associated with these are high fiber content and ANFs along with lower protein and AvP which restrict inclusion level in diet and turn it into a waste which impacts not only economic but also environmental (Kamboj et al. 2024). For overcoming the limitations involved in such by‐products, some physical, chemical, and biological processes have been developed by researchers.

Physical processing such as microwave treatment and extrusion are helpful in stabilizing rice bran (RB) by inactivating lipases and restraining rancidity (Irakli et al. 2020; Yılmaz Tuncel 2023; Lavanya et al. 2019) but do not improve nutritional bioavailability or reduce ANFs (Das et al. 2025). On the other hand, chemical processes like alkaline hydrolysis (NaOH, Ca(OH)_2_) and acid hydrolysis can improve digestibility and reduce ANFs to some extent (Ferris 2024). However, the use of such inorganic acids and bases is not always permitted considering the factor of food safety. Besides, microbial fermentation is also proven for increasing protein value, decreasing fiber content (Huervana et al. 2024; Islam et al. 2022), reducing ANFs like phytates and trypsin inhibitors, and also improves phosphorus availability (Liza et al. 2022; Seyoum et al. 2022).

Yeast fermentation with *Saccharomyces cerevisiae* fulfills various purposes like enhancement of taste, flavor, texture, nutritional value with ANFs reduction, fiber hydrolysis and enzyme release (e.g., phytases) (Rai and Jeyaram 2017). This process also directs to increased protein and reduction of fiber (Aktar et al. 2024; Ullah et al. 2021) and enrichment with bioactive phenolic compounds (Hettiarachchy 2015). Several studies reported positive effects of fermentation on RB, RP and DORB in different feed formulations (Tahir et al. 2025; Sharif et al. 2020; Su et al. 2022). Along with nutritional revalorization, these by‐products are promising substrates for bioethanol production, an integral component of sustainable energy strategies. Ethanol, being a clean‐burning and low‐carbon fuel in comparison with fossil fuels, has garnered global interest in the context of limiting greenhouse gases and decreasing import reliance on energy (Limayem and Ricke 2012; Balat and Balat 2009). Moreover, the manufacture of ethanol from rice by‐products follows the principles of circular economy policy and overall economic effectiveness of rice processing plants (Kamboj et al. 2024). Despite the advancement in bioconversion technologies, integrated studies that compare the chemical and nutritional alterations of RB, RP, and DORB under optimal yeast fermentation operating procedures are lacking. Moreover, little is known about how such enhanced qualities would enhance their value as functional ingredients in animal feed applications. This study therefore attempted to investigate the effect of solid‐state yeast fermentation on the chemical and functional characteristics of RB, RP, and DORB and find out the optimum fermentation conditions (yeast level, moisture level and time) to achieve maximum value as nutritionally enhanced, value‐added feed ingredients as well as ethanol production.

## 2 Materials and Methods

### 2.1 Experimental Design

The experiment was conducted to evaluate rice milling by‐products namely rice bran (RB), rice polish (RP), and de‐oiled rice bran (DORB) to evaluate the impact of moisture level and yeast inoculum concentration on the fermentation performance. A 2 × 3 × 9 factorial design was employed, consisting of two yeast doses (1.0% and 2.0%), three moisture levels (40%, 50%, 60%), and 9 different time0, 6, 12, 18, 24, 30, 36, 42, and 48 h of *Saccharomyces cerevisiae* under controlled laboratory conditions (39°C), each with three replications. Gas and ethanol production were used as indicators of fermentation kinetics and efficiency.

### 2.2 Fermentation Procedure

For each treatment, 30 g of the respective feed type (RB/RP/DORB) was taken and then yeast was added at levels 1.0% or 2.0% (w/w) of the total solution and mixed properly with the dry sample. The required amount of distilled water to maintain water and substrate ratio {2:3 (40%) or 1:1 (50%) or 3:2 (60%)} was added for achieving the desired moisture content, and the mixture was homogenized manually using a spoon. The amount of water required was calculated on a dry matter basis. After proper mixing, approximately 2–4 g of the prepared sample was placed into gas‐tight glass syringes. Then those syringes were incubated and gas production was recorded at 6‐h intervals up to 48 h. After ending the fermentation period, the fermented samples were discarded.

### 2.3 Gas Measurement and Calculation

Gas production was measured using a syringe‐based in vitro gas test (Rymer et al. 2005) and similar to Theodorou et al. (1994), with the cumulative gas volume (mL/100 g DM) recorded at 6‐h intervals. The amount of moles of CO_2_ production was calculated using the molar volume at room temperature and pressure (RTP, 24 L/mol). These moles directly correspond to ethanol production as a 1:1 value. Ethanol concentration (% w/v) was calculated as:

$$\%\text{Ethanol}\;\left(w/v\right)=\frac{\text{Moles of Ethanol}\times 46\;}{\text{Volume of Fermentation Medium}\;\left(\mathrm{ml}\right)}\times 100$$

This method aligns with standard bioethanol estimation techniques (York University 2013).

### 2.4 Chemical and Statistical Analysis

After fermentation, several samples (depending gas production value) were analyzed for dry matter, crude protein, crude fiber, ether extract, and ash following AOAC protocols (AOAC 2005). Metabolic Energy (ME) was calculated using the formula by Adeyina et al. (2017). Phosphorus fractions were determined via H_2_SO_4_ and H_2_O_2_ digestion followed by colorimetric detection at 660 nm for total‐phosphorus (ISO 1998). Phytate phosphorus was measured by using FeCl_3_ precipitation and subsequent digestion (Haug and Lantzsch 1983). Available phosphorus was computed as: Available P = Total P − Phytate P (Chowdhury and Koh 2019). Single effect was subjected to one‐way ANOVA and multiple effect (yeast, moisture, and time) were determined by three‐way ANOVA using IBM SPSS Statistics v.20 (IBM Crop), with significance determined at *p* < 0.05. Tukey's HSD test was employed for post hoc comparisons. The interaction effects of yeast dose, moisture level, and time were analyzed to identify the optimal fermentation conditions for maximizing gas output, ethanol yield, and nutrient availability.

## 3 Results

### 3.1 Fermentation of Rice Bran

#### 3.1.1 Fermentation Dynamics of Rice Bran

Rice bran showed a high fermentative capacity, with cumulative gas production (CGP) which was significantly affected by yeast dose, moisture level, and duration of fermentation. Surprisingly, increasing yeast dose from 1.0% to 2.0% CGP increased from 1540 to 2642 mL/100 g DM (Table 1), underlining yeast's role in enhancing microbial activity. The role of moisture level was also dominant, as maximum (*p* < 0.001) fermentation (2837 mL/100 g DM) was gained at 60% moisture, underlining the importance of enough hydration in supporting substrate accessibility and enzymatic action. The effect of incubation time markedly influenced the fermentation pattern. Among the nine different time points evaluated, cumulative gas production (CGP) increased significantly up to 12 h, beyond which the changes were only numerical, not statistical. Highly significant interactions (*p* < 0.001) among yeast, moisture, and time further highlight the need for synergistic optimization of these parameters to maximize fermentation efficiency in rice bran.

**TABLE 1:** Cumulative gas and ethanol production from fermented rice bran, rice polish and de‐oiled rice bran.

Treatment	CGP (mL/100 g DM)			CEP (mL/100 g DM)		
	Rice bran	Rice polish	De‐oiled rice bran	Rice bran	Rice polish	De‐oiled rice bran
Yeast level (%)						
1.0	1540 ± 35	1492 ± 35	285 ± 13	1.962 ± 0.066	1.313 ± 0.039	0.230 ± 0.012
2.0	2642 ± 35	1069 ± 35	106 ± 13	2.388 ± 0.066	0.692 ± 0.039	0.092 ± 0.012
Moisture level (%)						
40	959^c^ ± 43	827^b^ ± 43	25^b^ ± 16	1.187^c^ ± 0.081	0.906^b^ ± 0.048	0.022^b^ ± 0.015
50	2477^b^ ± 43	1444^a^ ± 43	52^b^ ± 16	2.504^b^ ± 0.081	0.885^b^ ± 0.048	0.044^b^ ± 0.015
60	2837^a^ ± 43	1572^a^ ± 43	510^a^ ± 16	2.834^a^ ± 0.081	1.216^a^ ± 0.048	0.418^a^ ± 0.015
Time (hour)						
0	00^c^ ± 00	00^f^ ± 00	00^c^ ± 00	00^c^ ± 00	00^e^ ± 00	00^b^ ± 00
6	1840^b^ ±75	510^e^ ±75	46^b^ ± 28	1.952^b^ ± 0.141	0.408^d^ ± 0.084	0.038^b^ ± 0.026
12	2311^a^ ±75	997^d^ ±75	185^a^ ± 28	2.426^ab^ ± 0.141	0.772^c^ ± 0.084	0.153^a^ ± 0.026
18	2475^a^ ±75	1327^cd^ ± 75	229^a^ ± 28	2.589^a^ ± 0.141	1.036b^c^ ± 0.084	0.190^a^ ± 0.026
24	2524^a^ ± 75	1528^bc^ ± 75	256^a^ ± 28	2.627^a^ ± 0.141	1.193^ab^ ± 0.084	0.221^a^ ± 0.026
30	2523^a^ ± 75	1689^ab^ ± 75	272^a^ ± 28	2.618^a^ ± 0.141	1.326^ab^ ± 0.084	0.224^a^ ± 0.026
36	2472^a^ ± 75	1778^ab^ ± 75	264^a^ ± 28	2.557^a^ ± 0.141	1.393^a^ ± 0.084	0.217^a^ ± 0.026
42	2393^a^ ± 75	1831^ab^ ± 75	258^a^ ± 28	2.463^ab^ ± 0.141	1.436^a^ ± 0.084	0.212^a^ ± 0.026
48	2280^a^ ± 75	1865^a^ ± 75	251^a^ ± 28	2.342^ab^ ± 0.141	1.459^a^ ± 0.084	0.206^a^ ± 0.026
SEM	25.1	24.92	9.33	0.047	0.028	0.009
Sources of variation (*p‐value*)						
Yeast	< 0.001	< 0.001	< 0.001	< 0.001	< 0.001	< 0.001
Moisture	< 0.001	< 0.001	< 0.001	< 0.001	< 0.001	< 0.001
Time	< 0.001	< 0.001	< 0.001	< 0.001	< 0.001	< 0.001
Yeast × Moisture	< 0.001	< 0.001	0.001	< 0.001	< 0.001	< 0.001
Yeast × Time	< 0.001	0.019	< 0.001	0.770	< 0.001	0.009
Time × Moisture	< 0.001	< 0.001	< 0.001	< 0.001	0.961	< 0.001
Yeast × Moisture × Time	< 0.001	0.001	< 0.001	0.732	0.003	< 0.001
Sample size						
Yeast	81	81	81	81	81	81
Moisture level	54	54	54	54	54	54
Time	18	18	18	18	18	18

*Note:* ^a,b,c,d,e,f^: The differences between the averages shown with different superscripts on the same line are statistically significant (Mean ± Standard Error).

Abbreviations: CEP, cumulative ethanol production; CGP, cumulative gas production.

#### 3.1.2 Effect of Moisture

Gas production in RB was highly significant with moisture level (*p* < 0.001) where 60% moisture produced highest values across all incubation periods in comparison with 40% and 50% (Table 2). At the early stage (6 h), most of the fermentation was recorded at 60% moisture with 2% yeast (2932 mL/100 g DM), followed by 50% (2381 mL) and 40% (1614 mL). At 12 h, gas production became lower compared to 6 h in all moisture levels (805 mL, 1082 mL, and 117 mL for 60%, 50%, and 40% moisture, respectively). Beyond 18 and 36 h, gas output dropped at 40% and 50% moisture levels. Despite this overall decline, the superiority of the 60% moisture group was maintained throughout the fermentation process. Additionally, highly significant interaction among yeast, moisture, and time (*p* < 0.001; Table 1) suggested that CGP was highly influenced by the combined effects of all factors over time.

**TABLE 2:** Gas production of fermented rice bran at different moisture levels with 2% yeast.

Time (h)	GP at different moisture on 6 h interval				Time (h)	Cumulative GP (CGP) at different moisture			
	40%	50%	60%	*p*		40%	50%	60%	*p*
0	00 ± 0.0	00 ± 0.0	00 ± 0.0	—	0	0 ± 0.0	0 ± 0.0	0 ± 0.0	—
0–6	1614^b^ ± 343	2381^ab^ ±332	2932^a^ ± 98	0.044	0–6	1613^b^ ± 343	2381^ab^ ±332	2932^a^ ± 98	0.004
6–12	117^b^ ± 126	1082^a^ ± 109	805^a^ ± 51	0.001	0–12	1731^b^ ± 270	3463^a^ ± 223	3736^a^ ± 149	0.001
12–18	−301^b^ ± 186	391^a^ ± 78	466^a^ ± 82	0.010	0–18	1430^b^ ± 231	3854^a^ ± 150	4203^a^ ± 67	< 0.001
18–24	−223^b^ ± 133	290^a^ ± 137	115^a^ ± 65	0.053	0–24	1207^b^ ± 170	4114^a^ ± 29	4317^a^ ± 130	< 0.001
24–30	−243^b^ ± 83	86^a^ ± 46	102^a^ ± 13	0.008	0–30	964^b^ ± 185	4201^a^ ± 46	4419^a^ ± 125	< 0.001
30–36	−251^b^ ± 83	−101^ab^ ± 60	65^a^ ± 14	0.027	0–36	712^b^ ± 159	4100^a^ ± 85	4484^a^ ± 137	< 0.001
36–42	−157 ± 70	−207 ± 119	60 ± 2	0.115	0–42	555^c^ ± 89	3892^b^ ± 189	4544^a^ ± 138	< 0.001
42–48	−54 ± 34	−450 ± 298	37 ± 10	0.188	0–48	501^c^ ± 55	3442^b^ ± 342	4581^a^ ± 134	< 0.001

*Note:* ^a,b,c^: The differences between the averages shown with different superscripts on the same line are statistically significant (Mean ± Standard Error).

Abbreviations: CGP, cumulative gas production; GP, gas production.

#### 3.1.3 Effect of Yeast Concentration

Rice bran fermentation was strongly influenced by yeast dose. Increasing yeast dose from 1% to 2% noticeable enhancement of gas production occurred by rising from 1875 to 2932 mL/100 g DM within just 6 h (Table 3). The higher yeast dose (2%) also maintained longer activity by still producing 37 mL at 48 h whereas the 1% yeast dose declined to (−34) mL over the same period of time. These results clearly demonstrate the superior efficiency of 2% yeast in accelerating and maintaining microbial fermentation activity in rice bran. Statistically, the effect of yeast was highly significant (*p* < 0.001), confirming its critical role in fermentation performance (Table 1).

**TABLE 3:** Gas production of fermented RB, RP and DORB at 60% moisture level at 6 h interval with 1% and 2% yeast.

Time (h)	0	0–6	6–12	12–18	18–24	24–30	30–36	36–42	42–48
Yeast	*Rice bran*								
1%	00 ± 0.0	1875 ± 126	194 ± 72	146 ± 75	43 ± 30	59 ± 16	65 ± 22	7 ± 8	−34 ± 34
2%	00 ± 0.0	2932 ± 98	805 ± 51	466 ± 82	115 ± 65	102 ± 13	65 ± 14	60 ± 2	37 ± 10
*p‐value*	—	0.003	0.002	0.045	0.372	0.106	0.987	0.003	0.114
Yeast	*Rice polish*								
1%	00 ± 0.0	693 ± 104	486 ± 52	443 ± 29	205 ± 36	218 ± 46	91 ± 18	53 ± 34	62 ± 37
2%	00 ± 0.0	552 ± 105	713 ± 40	395 ± 38	267 ± 22	147 ± 40	134 ± 31	83 ± 4	81 ± 17
*p‐value*	—	0.395	0.026	0.379	0.221	0.307	0.295	0.438	0.654
Yeast	*De‐oiled rice bran*								
1%	00 ± 0.0	119 ± 36	568 ± 63	214 ± 22	143 ± 20	94 ± 31	6 ± 28	00 ± 00	00 ± 00
2%	00 ± 0.0	50 ± 13	178 ± 164	49 ± 29	20 ± 5	03 ± 23	−45 ± 51	−24 ± 30	−14 ± 14
*p‐value*	—	0.145	0.09	0.01	0.004	0.077	0.428	0.469	0.374

*Note:* ^a,b,c^: The differences between the averages shown with different superscripts on the same line are statistically significant (Mean ± Standard Error).

#### 3.1.4 Effect of Time

The effect of incubation time on CGP from RB was pronounced during the early phase of fermentation. At 6 h, CGP increased to 1840 mL/100 g DM (Table 1) which showed an intense onset of microbial activity. By 12 h, CGP reached its highest value of 2311 mL/100 g DM which indicated that the majority of fermentable substrates were rapidly utilized within this period. After 12 h, the tendency shifted as the values continued to rise only numerically with CGP ranging from 2475 to 2524 mL/100 g DM between 18 and 24 h but non‐significantly compared to the 12‐h peak. Beyond 30 h, GP started to show a gradual decline, with values decreasing to 2472 mL/100 g DM at 36 h and further down to 2280 mL/100 g DM at 48 h (Table 1). Overall, the data suggested that RB fermentation is mostly active within the first 12 h and then gas production does not follow any significant role. The statistical significance (*p* < 0.001) underscores time as an important factor influencing CGP.

#### 3.1.5 Effect on Chemical Composition

Rice bran showed highly response to fermentation with significant changes of nutritional composition across multiple parameters. Notably, crude protein increased by 23.1% (Table 4) (14.67%–18.06%, *p* < 0.001) by driven microbial protein and carbohydrate degradation. In contrast, crude fiber declined by 20.1% (*p* < 0.001) which reflecting improved digestibility. The energy‐related fractions were also highly affected. Ether extract nearly doubled (15.07%–29.73%, *p* < 0.001), while nitrogen‐free extract dropped from 35.47% to 19.8% (*p* < 0.001), indicating intense microbial activity. These changes enhance a 25.3% rise in metabolizable energy (*p* < 0.001). Available phosphorus increased by 55.6% (0.27–0.42, *p* = 0.003), while phytate phosphorus decreased by 9% (0.80–0.73, *p* = 0.001), shifting the PP:AvP ratio from 75%:25% to 64%:36% (Table 4). The combination effects of those interactions reported fermentation as a great strategy to enrich nutrient density and enhance mineral accessibility in rice bran.

**TABLE 4:** Chemical composition of fermented rice bran with 60% moisture and 2.0% yeast at 0, 24, and 48 h.

Parameter	RB‐0	RB‐24	RB‐48	*p*
Dry matter	100 ± 00	100 ± 00	100 ± 00	—
Crude protein	14.67^b^ ± 0.34	17.01^a^ ± 0.27	18.06^a^ ± 0.18	< 0.001
Crude fiber	23.04^a^ ± 0.64	18.81^b^ ± 0.02	18.41^b^ ± 0.1	< 0.001
Ether extract	15.07^c^ ± 0.34	19.98^b^ ± 0.16	29.73^a^ ± 0.89	< 0.001
Ash	11.74^b^ ± 0.77	13.47^ab^ ± 0.1	13.99^a^ ± 0.41	0.03
Nitrogen free extract	35.47^a^ ± 0.79	30.73^b^ ± 0.54	19.8^c^ ± 1.1	< 0.001
ME (kcal/kg)	3035^c^ ± 12	3355^b^ ± 4	3803^a^ ± 40	< 0.001
Total phosphorus	1.07^b^ ± 0.005	1.1^ab^ ± 0.01	1.14^a^ ± 0.01	0.008
Phytate phosphorus (PP)	0.8^a^ ± 0.006	0.75^b^ ± 0.01	0.73^b^ ± 0.01	0.001
Available phosphorus (AvP)	0.27^b^ ± 0.01	0.35^a^ ± 0.02	0.42^a^ ± 0.02	0.003
PP: AvP	75:25	68:32	64:36	—

*Note:* ^a,b,c^: The differences between the averages shown with different superscripts on the same line are statistically significant (Mean ± Standard Error).

Abbreviation: RB, rice bran.

#### 3.1.6 Ethanol Production

RB produced the highest ethanol among the substrates where performance depended on yeast, moisture, and incubation period (time). By increasing yeast dose from 1% to 2%, overall ethanol rose from 1.96 to 2.39 g/100 mL (Table 1). Moisture has a dominant role because the maximum yields were consistently achieved at 60% moisture. At this level, ethanol peaked at 3.42 g/100 mL with 1% yeast and 3.49 g/100 mL with 2% yeast (Table 5), both significantly higher than lower moisture (40% and 50%) treatments. With 2% yeast and 60% moisture, time also influenced fermentation patterns, resulting in ethanol accumulation of 2.85 g/100 mL in just 12 h and continued to increase until 48 h. These results highlight that rice bran responds strongly to both yeast dose and moisture availability, where optimal conditions were 2% yeast and 60% moisture, sustaining higher ethanol production throughout the fermentation period.

**TABLE 5:** Cumulative ethanol production of RB, RP and DORB at 40%, 50% and 60% moisture with 1% and 2% yeast (g/100 mL).

Time (h)	0–6	0–12	0–18	0–24	0–30	0–36	0–42	0–48
*Moisture level (%) yeast level (1.0%)*								
Rice bran								
40%	1.21 ± 0.10	1.46 ± 0.10	1.6 ± 0.21	1.51 ± 0.27	1.38 ± 0.34	1.22 ± 0.32	0.98^c^ ± 0.27	0.78^b^ ± 0.19
50%	1.32 ± 0.44	1.81 ± 0.30	2.06 ± 0.19	2.24 ± 0.14	2.37 ± 0.86	2.44 ± 0.08	2.46^ab^ ± 0.08	2.47^ab^ ± 0.13
60%	2.61 ± 0.47	2.92 ± 0.59	3.18 ± 0.74	3.26 ± 0.81	3.36 ± 0.85	3.46 ± 0.88	3.46^a^ ± 0.87	3.42^a^ ± 0.87
*p‐value*	**0.071**	**0.083**	**0.116**	**0.116**	**0.1**	**0.071**	**0.043**	**0.03**
Rice polish								
40%	0.63 ± 0.26	1.15 ± 0.24	1.65 ± 0.29	1.93 ± 0.31	2.21 ± 0.34	2.31 ± 0.31	2.39 ± 0.26	2.38 ± 0.18
50%	0.34 ± 0.12	0.63 ± 0.09	0.87 ± 0.10	1.00 ± 0.11	1.13 ± 0.11	1.20 ± 0.12	1.22 ± 0.13	1.24 ± 0.15
60%	0.66 ± 0.16	1.10 ± 0.19	1.52 ± 0.26	1.72 ± 0.32	1.92 ± 0.37	2.01 ± 0.39	2.07 ± 0.44	2.14 ± 0.48
*p‐value*	**0.464**	**0.167**	**0.107**	**0.107**	**0.098**	**0.088**	**0.079**	**0.087**
De‐oiled rice bran								
40%	0.016^b^ ± 0.008	0.016^b^ ± 0.008	0.016^b^ ± 0.008	0.012^b^ ± 0.007	0.012^b^ ± 0.007	0.012^b^ ± 0.007	0.012^b^ ± 0.007	0.008^b^ ± 0.008
50%	0.015^b^ ± 0.003	0.033^b^ ± 0.016	0.033^b^ ± 0.016	0.03^b^ ± 0.013	0.03^b^ ± 0.013	0.026^b^ ± 0.009	0.026^b^ ± 0.009	0.019^b^ ± 0.004
60%	0.095^a^ ± 0.028	0.56^a^ ± 0.088	0.73^a^ ± 0.102	0.84^a^ ± 0.105	0.92^a^ ± 0.090	0.92^a^ ± 0.111	0.92^a^ ± 0.111	0.92^a^ ± 0.111
*p‐value*	**0.024**	**< 0.001**	**< 0.001**	**< 0.001**	**< 0.001**	**< 0.001**	**< 0.001**	**< 0.001**
*Moisture level (%) yeast level (2.0%)*								
Rice bran								
40%	2.09 ± 0.44	2.23^b^ ± 0.31	1.83^b^ ± 0.22	1.55^c^ ± 0.16	1.24^b^ ± 0.21	0.92^b^ ± 0.20	0.72^b^ ± 0.11	0.65^b^ ± 0.07
50%	2.24 ± 0.22	3.28^a^ ± 0.12	3.66^a^ ± 0.12	3.91^a^ ± 0.18	4^a^ ± 0.21	3.9^a^ ± 0.15	3.69^a^ ± 0.06	3.25^a^ ± 0.22
60%	2.24 ± 0.07	2.85^ab^ ± 0.07	3.21^a^ ± 0.15	3.29^b^ ± 0.11	3.37^a^ ± 0.12	3.42^a^ ± 0.11	3.47^a^ ± 0.11	3.49^a^ ± 0.12
*p‐value*	**0.915**	**0.025**	**0.001**	**< 0.001**	**< 0.001**	**< 0.001**	**< 0.001**	**< 0.001**
Rice polish								
40%	0.21 ± 0.10	0.27^b^ ± 0.12	0.25^b^ ± 0.10	0.23^b^ ± 0.08	0.20^b^ ± 0.05	0.18^b^ ± 0.02	0.17^b^ ± 0.01	0.16^b^ ± 0.01
50%	0.28 ± 0.11	0.72^a^ ± 0.17	0.92^a^ ± 0.17	1.10^a^ ± 0.22	1.22^a^ ± 0.26	1.31^a^ ± 0.29	1.37^a^ ± 0.30	1.38^a^ ± 0.31
60%	0.33 ± 0.04	0.76^a^ ± 0.05	1.01^a^ ± 0.08	1.17^a^ ± 0.09	1.27^a^ ± 0.12	1.35^a^ ± 0.14	1.40^a^ ± 0.15	1.45^a^ ± 0.16
*p‐value*	**0.653**	**0.049**	**0.009**	**0.007**	**0.006**	**0.006**	**0.006**	**0.007**
De‐oiled rice bran								
40%	0.037 ± 0.01	0.037 ± 0.01	0.037 ± 0.01	0.037 ± 0.01	0.037 ± 0.01	0.034 ± 0.01	0.034 ± 0.01	0.031 ± 0.01
50%	0.03 ± 0.01	0.08 ± 0.02	0.08 ± 0.02	0.08 ± 0.02	0.09 ± 0.02	0.08 ± 0.02	0.07 ± 0.02	0.067 ± 0.03
60%	0.04 ± 0.01	0.2 ± 0.15	0.24 ± 0.18	0.26 ± 0.18	0.26 ± 0.16	0.22 ± 0.12	0.20 ± 0.10	0.19 ± 0.08
*p‐value*	**0.475**	**0.462**	**0.399**	**0.345**	**0.275**	**0.209**	**0.15**	**0.123**

*Note:* ^a,b,c^: The differences between the averages shown with different superscripts on the same line are statistically significant (Mean ± Standard Error).

### 3.2 Fermentation of Rice Polish

#### 3.2.1 Fermentation Dynamics of Rice Polish

Rice polish demonstrated an intermediate fermentation capacity compared with rice bran and de‐oiled rice bran. Cumulative gas production (CGP) was significantly affected by yeast dose, substrate moisture level, and incubation time (Table 1). Interestingly, lower inoculation (1% yeast) supported greater gas production (1492 mL/100 g DM) than 2% yeast (1069 mL/100 g DM; *p* < 0.001), suggesting that excessive inoculum may have created metabolic imbalance or substrate competition. Moisture content played a decisive role, with maximum gas release achieved at 60% (1572 mL/100 g DM; *p* < 0.001). Gas production in rice polish increases rapidly during the first 12 h and continues to increase up to 48 h. Unlike rice bran and de‐oiled rice bran, negative values were not observed (Table 6) that reflect a more stable and sustained fermentation profile. Strong interaction effects (*p* < 0.001) emphasize that RP fermentation is highly sensitive to the balance of microbial load, moisture, and incubation duration.

**TABLE 6:** Gas production of fermented rice polish at different moisture levels with 2% yeast.

Time (h)	GP at different moisture on 6 h interval				Time (h)	Cumulative GP (CGP) at different moisture			
	40%	50%	60%	*p*		40%	50%	60%	*p*
0	00 ± 0	00 ± 0	00 ± 0	—	0	0 ± 0	0 ± 0	0 ± 0	—
0–6	255 ± 147	402 ± 154	552 ± 105	0.374	0–6	255 ± 147	402 ± 154	552 ± 105	0.374
6–12	67^b^ ± 40	689^a^ ± 85	713^a^ ± 40	< 0.001	0–12	322^b^ ± 176	1091^a^ ± 215	1265^a^ ± 130	0.02
12–18	−23^b^ ± 23	308^a^ ± 33	395^a^ ± 38	< 0.001	0–18	299^b^ ± 154	1398^a^ ± 209	1660^a^ ± 105	0.002
18–24	−21^b^ ± 22	286^a^ ± 84	267^a^ ± 22	0.010	0–24	278^b^ ± 134	1684^a^ ± 293	1927^a^ ± 84	0.002
24–30	−45^b^ ± 40	168^a^ ± 40	147^a^ ± 40	0.018	0–30	234^b^ ± 94	1852^a^ ± 329	2074^a^ ± 82	0.001
30–36	−38^b^ ± 44	130^a^ ± 43	134^a^ ± 31	0.036	0–36	196^b^ ± 50	1982^a^ ± 369	2208^a^ ± 69	0.001
36–42	−14^b^ ± 14	94^a^ ± 24	83^a^ ± 4	0.006	0–42	182^b^ ± 37	2076^a^ ± 393	2291^a^ ± 72	0.001
42–48	−05^b^ ± 5	18^b^ ± 18	81^a^ ± 17	0.014	0–48	178^b^ ± 32	2094^a^ ± 411	2372^a^ ± 89	0.001

*Note:* ^a,b^: The differences between the averages shown with different superscripts on the same line are statistically significant (Mean ± Standard Error).

Abbreviations: CGP, cumulative gas production; GP, gas production.

#### 3.2.2 Effect of Moisture

Availability of moisture strongly influenced the fermentation efficiency of rice polish. At 12 h, gas production reached its peak with 60% moisture (713 mL/100 g DM), closely followed by 50% (689 mL), while fermentation was minimal at 40% (67 mL; *p* < 0.001) (Table 6). After this point, gas production declined progressively across all treatments. By 24 h, it dropped to 267 and 286 mL for 60% and 50% moisture, respectively, while the 40% treatment showed a negative output (−21 mL) that indicated microbial stress under restricted hydration. CGP over 48 h confirmed the excellence of 60% moisture by yielding 2372 mL in comparison with 2094 mL at 50% and only 178 mL at 40% (Table 6). Significant yeast × moisture and time × moisture interactions (*p* < 0.001) highlight that substrate hydration not only supports higher fermentation peaks but also sustains microbial activity for longer periods.

#### 3.2.3 Effect of Yeast Concentration

Although overall fermentation favored 1% yeast, when interacting with higher moisture levels (60%), then 2% showed better performance patterns than 1% dose (Table 3). At 60% moisture, 2% yeast produced higher peak gas (713 mL/100 g DM at 12 h) and sustained activity through 48 h (81 mL), compared with 486 mL and 62 mL under 1% yeast (Table 3). The cumulative production was also higher with 2% yeast (2372 mL vs. 2251 mL). The results suggest that while excessive doses may decrease efficiency in low‐moisture environments, at 60% moisture the higher yeast dose (2%) accelerated substrate colonization and confirmed more consistent fermentation. Yeast and its interactions with moisture and time were all highly significant (*p* < 0.001), confirming its central role in fermentation performance.

#### 3.2.4 Effect of Time

The time sequence of fermentation in rice polish revealed a more gradual and stable route than rice bran. Gas production increases rapidly to 510 mL/100 g DM within 6 h and then continued to increase to 997 mL at 12 h that marking the most active phase of fermentation (Table 1). Unlike RB and DORB, RP did not exhibit negative gas production values at later stages, which suggests that its nutrient profile supported prolonged microbial activity. Cumulative gas production rose consistently throughout the 48‐h period, reaching 1865 mL. This extended fermentation window indicates that RP provides a balanced supply of fermentable substrates that supports both rapid early growth and stable microbial metabolism over time.

#### 3.2.5 Effect on Chemical Composition

Fermentation markedly improved the nutritional profile of rice polish. Crude fiber declined significantly by 18.2% (8.12–6.64; *p* =0.004) that reflecting effective fiber degradation. Besides, ether extract increased by 66% (9.61%–15.96%; *p* =0.001) which suggested release of bound lipids and potential microbial lipid synthesis (Table 7). Protein content remained almost stable (11.65–12.15; *p* = 0.564) but metabolizable energy increased by 8.8% (3256 kcal/kg–3543 kcal/kg; *p* = 0.001) that underscored improved energy density. Phosphorus dynamics were particularly favorable while total P increased from 1.81 to 1.98 (*p* = 0.003) and available P rose from 0.95 to 1.26 (*p* = 0.015) accompanied by a shift in the PP:AvP ratio from 48:52 to 36:64. These improvements highlight RP's enhanced digestibility and mineral utilization following fermentation, making it a more efficient feed ingredient for non‐ruminants.

**TABLE 7:** Chemical composition of fermented rice polish with 60% moisture and 2% yeast at 0, 30, and 48 h.

Parameter	RP‐0	RP‐30	RP‐48	*p*
Dry matter	100 ± 00	100 ± 00	100 ± 00	—
Crude protein	11.65 ± 0.8	12.41 ± 0.03	12.15 ± 0.24	0.564
Crude fiber	8.12^a^ ± 0.18	7.66^a^ ± 0.13	6.64^b^ ± 0.24	0.004
Ether extract	9.61^c^ ± 0.62	12.02^b^ ± 0.34	15.96^a^ ± 0.87	0.001
Ash	13.2 ± 0.71	13.95 ± 0.06	14.89 ± 0.46	0.127
Nitrogen free extract	57.43^a^ ± 2.3	53.95^ab^ ± 0.23	50.36^b^ ± 1.12	0.042
ME (kcal/kg)	3256^c^ ± 2	3358^b^ ± 18	3543^a^ ± 46	0.001
Total phosphorus	1.81^b^ ± 0.03	1.89^ab^ ± 0.01	1.98^a^ ± 0.003	0.003
Phytate phosphorus (PP)	0.86 ± 0.05	0.78 ± 0.01	0.72 ± 0.03	0.07
Available phosphorus (AvP)	0.95^b^ ± 0.07	1.11^ab^ ± 0.02	1.26^a^ ± 0.03	0.015
PP: AvP	48:52	41:59	36:64	—

*Note:* ^a,b,c^: The differences between the averages shown with different superscripts on the same line are statistically significant (Mean ± Standard Error).

#### 3.2.6 Ethanol Production

Ethanol production reached 2.14 g/100 mL within 48 h (*p* = 0.087; Table 5) at 1% yeast and 60% moisture. On the other hand, 2% yeast with the same moisture level resulted in lower ethanol production by reaching only 1.45 g/100 mL (*p* = 0.007; Table 5) that was possibly due to early substrate depletion or inhibitory metabolite accumulation. Despite these differences, RP demonstrated reliable fermentability and ethanol production, which positioned it as a dual‐purpose substrate and capable of enhancing feed value while also contributing to small‐scale bioethanol production under optimized conditions.

### 3.3 Fermentation of De‐Oiled Rice Bran

#### 3.3.1 Fermentation Dynamics of De‐Oiled Rice Bran

De‐oiled rice bran (DORB) showed the lowest fermentative capacity among the three substrates, with cumulative gas production (CGP) and ethanol yield markedly lower than rice bran and rice polish (Table 1). 2% yeast dose resulted in reduced gas output compared with 1% yeast (106 vs. 285 mL/100 g DM; *p* < 0.001), which suggests substrate limitation or microbial inhibition under excessive inoculation level. Moisture played an important compensatory role with 60% moisture by gas output (510 mL/100 g DM; *p* < 0.001). Highest fermentation occurred at 12 h and then gas production declined, which turned negative by 36 h. Despite these effects, fermentation efficiency of DORB remained limited, suggesting its lower availability of fermentable carbohydrates. The strong statistical significance of all interactive effects (*p* < 0.001) supports that precise calibration of yeast, moisture, and incubation time is critical to unlock the limited fermentative potential of de‐oiled rice bran.

#### 3.3.2 Effect of Moisture

Moisture availability showed a noticeable influence on DORB fermentation. At low moisture levels (40% and 50%), gas output was negligible but 60% consistently supported higher activity, peaking within the first 12 h (Table 8). However, this was not continued as values declined steadily thereafter and became negative beyond 30 h. High significant value among yeast, moisture and time interactions (*p* < 0.001) confirms that proper moisture level temporarily improves fermentability in DORB but cannot fully compensate for its nutrient limitations.

**TABLE 8:** Gas production of fermented de‐oiled rice bran at different moisture levels with 2% yeast.

Time (h)	GP at different moisture on 6 h interval				Time (h)	Cumulative GP (CGP) at different moisture			
	40%	50%	60%	*p*		40%	50%	60%	*p*
0	00 ± 0	00 ± 0	00 ± 0	—	0	0 ± 0	0 ± 0	0 ± 0	—
0–6	43 ± 10	31 ± 7	50 ± 13	0.469	0–6	43 ± 10	31 ± 7	50 ± 13	0.469
6–12	00 ± 00	61 ± 20	178 ± 164	0.455	0–12	43 ± 10	92 ± 26	228 ± 175	0.467
12–18	00 ± 00	04 ± 4	49 ± 29	0.157	0–18	43 ± 10	96 ± 26	276 ± 204	0.405
18–24	00 ± 00	04^b^ ± 3	20^a^ ± 5	0.014	0–24	43 ± 10	100 ± 23	297 ± 204	0.352
24–30	00 ± 6	04 ± 4	03 ± 23	0.975	0–30	43 ± 16	104 ± 26	300 ± 182	0.279
30–36	−03 ± 3	−04 ± 4	−45 ± 51	0.555	0–36	40 ± 13	99 ± 28	255 ± 131	0.210
36–42	00 ± 0	−12 ± 1	−24 ± 30	0.642	0–42	40 ± 13	88 ± 28	231 ± 101	0.148
42–48	−3 ± 3	−08 ± 4	−14 ± 14	0.682	0–48	37 ± 10	80 ± 32	217 ± 87	0.121

*Note:* ^a,b^: The differences between the averages shown with different superscripts on the same line are statistically significant (Mean ± Standard Error).

Abbreviations: CGP, cumulative gas production; GP, gas production.

#### 3.3.3 Effect of Yeast Concentration

In contrast to RB and RP where higher inoculum generally improved performance, DORB responded better to a lower (1%) yeast dose. At1% yeast dose, fermentation was more durable and reaching its peak within 12 h then shaped gradually by 48 h. Whereas 2% yeast dose caused an early wave followed by rapid collapse and negative values (Table 3). This pattern suggests that substrate insufficiency and osmotic stress limited microbial proliferation at higher inoculum levels. Statistically, yeast concentration and its interactions with moisture and time were all significant (*p* < 0.001), indicating that fermentation outcomes in DORB are highly dose‐sensitive.

#### 3.3.4 Effect of Time

In DORB, the effect of incubation time was obvious but far less stable than in RB or RP. Fermentation activity increased during the initial period by reaching its highest intensity within 12 h and then the process quickly lost momentum. The decline was progressive, with gas output falling sharply after 18 h and eventually turning negative beyond 30 h (Table 1). Unlike rice polish, which maintained stable fermentation throughout the 48‐h period, DORB exhibited only a brief window of activity, emphasizing its limited carbohydrate reserves and poor capacity to sustain prolonged microbial metabolism.

#### 3.3.5 Effect on Chemical Composition

Despite its low fermentability, DORB experienced substantial nutritional upgrading through yeast fermentation. Crude protein increased by 15.6% (*p* = 0.023), crude fiber declined by 18.9% (*p* = 0.003), and ether extract rose dramatically by 294% (*p* = 0.008) (Table 9). These changes converted into a 21% improvement in metabolizable energy (*p* = 0.005). Phosphorus level also improved significantly where available P rose from 0.82 to 1.02 (*p* < 0.001) and PP:AvP ratio shifted from 46:54 to 39:61. This finding indicates improved bioavailability despite only minor reductions in phytate P. Collectively, these enhancements confirm the potential of fermentation to upgrade DORB as a feed ingredient, even under conditions of limited fermentability.

**TABLE 9:** Chemical composition of fermented de‐oiled rice bran with 60% moisture and 1% yeast at 0, 30 and 48 h.

Parameter	DORB‐0	DORB‐30	DORB‐48	*p*
Dry matter	100 ± 00	100 ± 00	100 ± 00	—
Crude protein	11.72^b^ ± 0.08	12.78^ab^ ± 0.49	13.55^a^ ± 0.30	0.023
Crude fiber	22.06^a^ ± 0.06	20.3^a^ ± 0.33	17.89^b^ ± 0.77	0.003
Ether extract	2.74^b^ ± 0.4	6.03^ab^ ± 0.73	10.8^a^ ± 1.86	0.008
Ash	15.49^b^ ± 0.03	16.6^a^ ± 0.43	16.48^ab^ ± 0.49	0.04
Nitrogen free extract	47.99^a^ ± 0.44	44.24^ab^ ± 1.74	41.26^b^ ± 1.49	0.034
ME (kcal/kg)	2362^b^ ± 20	2537^b^ ± 18	2851^a^ ± 112	0.005
Total phosphorus	1.51^c^ ± 0.009	1.61^b^ ± 0.006	1.68^a^ ± 0.01	< 0.001
Phytate phosphorus (PP)	0.69^a^ ± 0.008	0.68^a^ ± 0.003	0.66^b^ ± 0.003	0.01
Available phosphorus (AvP)	0.82^c^ ± 0.016	0.93^b^ ± 0.008	1.02^a^ ± 0.014	< 0.001
PP: AvP	46:54	42:58	39:61	—

*Note:* ^a,b,c^: The differences between the averages shown with different superscripts on the same line are statistically significant (Mean ± Standard Error).

#### 3.3.6 Ethanol Production

DORB generate lowest ethanol among all by‐products, where ethanol production value was only 0.92 g/100 mL at 48 h with 1% yeast and 60% moisture (*p* < 0.001; Table 5) while 2% yeast produced even less (0.19 g/100 mL; Table 5). These limited outputs suggested that nutrient depletion and poor fermentable carbohydrate availability severely constrain DORB's role as a bioethanol substrate. Nevertheless, the marked improvements in proximate composition highlight its greater value for feed applications rather than for energy production.

## 4 Discussion

The study examined the impact of yeast fermentation ( *Saccharomyces cerevisiae* ) on the nutritional enhancement, fermentability, and bioavailability of phosphorus of rice milling by‐products: rice bran (RB), Rice Polish (RP) and de‐oiled rice bran (DORB).

### 4.1 Fermentation Efficiency of RB

Rice bran was demonstrated to be the optimal substrate for fermentation, highlighting the influence of composition and fermentative conditions on the key results such as nutritional quality, kinetics of fermentation, and phosphorus availability. High cumulative gas production results from both the abundance of readily fermentable sugars and the competency of yeast colonization found in the rice bran (Williams et al. 2005; Zhu et al. 2021). This is also in agreement with Erten et al. (2006), who said that a higher level of inoculum accelerates microbial colonization and the release of enzymes and therefore fermentation kinetics. However, the sharp spike at 6–12 h followed by a drop indicates that RB can promote rapid microbial growth in the phase of exponential growth but also experiences substrate limitation later (Russell 2003).

Ethanol production in RB closely paralleled gas release, reaching maximum at 48 h under optimal conditions, and thus RB is the best substrate for bioethanol. The strong correlation between CO_2_ production and ethanol yield confirms the use of gas production as a valid proxy measure of ethanol production (York University 2013; Azhar et al. 2017). The sustained ethanol production even at 30 h and later shows that, while basic carbohydrates were exhausted at an early stage, secondary fermentation of complex carbohydrates continued (Walker and Stewart 2016). The continued fermentation definite RB's superior nutritional value with both soluble and structural carbohydrates being accessible for microbial metabolism (Sivamaruthi et al. 2018). Compared to RP and DORB, the better performance of RB suggested its use as a feed ingredient and renewable bioethanol substrate under circular bio‐economy which is the recent consideration and agreed by other researchers (Nidhishree et al. 2024).

### 4.2 Fermentation Efficiency of Rice Polish (RP)

Rice polish (RP) was moderately but more stable in fermentation dynamics. Unlike the initial intense and fast activity of RB, RP supported slow but sustained fermentation. Interestingly, gas production was higher at 1.0% yeast compared to 2.0%, suggesting that excessive inoculum may have created competition for substrates, osmotic stress, or accumulation of inhibitory metabolites. These findings are consistent with earlier research where peak inoculum rather than maximum inoculum established effective fermentation (Nahak et al. 2023). Similar findings have been reported in solid‐state fermentation studies where optimal inoculum levels maximized microbial colonization and enzymatic activity while higher doses reduced production due to metabolic inhibition (Erten et al. 2006; Sivamaruthi et al. 2018).

Water level continued to be the deciding factor. At 60% hydration, the maximum gas production was at 12 h, and overall gas until 48 h with no negative values. This negated RB and DORB where a negative output was recorded under low hydration or where delayed stages due to microbial depression. RP's typical fermentation profile reveals the composition of nutrients in the RP favors long‐term microbial activity though at oppressed level relative to RB (Ahmad and Munaim 2017). Ethanol generation was similarly modest at 1.0% yeast and 60% water content which poses RP in the role for twin purpose utilization in both improving feed energy value and enabling low‐scale bioethanol production (van Dyk et al. 2025).

### 4.3 Fermentation Efficiency of De‐Oiled Rice Bran (DORB)

De‐oiled rice bran showed the worst fermentation tendency with gas and ethanol yield significantly lower than RB and RP. The highest cumulative gas yield (510 mL/100 g DM at 12 h with 2% yeast, 60% moisture) was not sustained, and values were negative at 30 h. Ethanol production was also low (0.92 g/100 mL at 48 h with 1.0% yeast), which corresponds to the low fermentable carbohydrate content in DORB. This concurs with Rathod and Srinivas (2018), who asserted that de‐oiling decreases energy substrates and increases anti‐nutritional factors and consequently hinders microbial metabolism.

Interestingly, 1.0% decreased dose gave more reproducible results than 2.0%. At the larger inoculum, osmotic shock and total nutrient starvation likely caused early disruption of microbial metabolism, which is characteristic of substrate–inoculum imbalance. Nahak et al. (2023) also reported the same, wherein excess inoculum was found to promote metabolic stress and reduced fermentation capacity.

The results emphasize that DORB requires substrate‐specific optimization, i.e., co‐fermentation with carbohydrate material or enzyme supplementation, to reach its potential in bioethanol applications. The identical approaches—i.e., supplementation of lignocellulose residues with simple sugars or the addition of hydrolytic enzymes—have been shown to increase the fermentability of low‐carbohydrate substrates (Sindhu et al. 2016). Nevertheless, in spite of reduced fermentability, the reported nutritional improvements after fermentation indicate that DORB is still possibly upgradeable for use in feed applications (Sivamaruthi et al. 2018; Nahak et al. 2023).

### 4.4 Nutritional Improvement Over Substrates

The most valuable outcome aside from fermentation kinetics was the dramatic increase in content of nutrients in all substrates. For RB, crude protein increased by 23.1%, crude fiber decreased by 20.1%, and ether extract increased by nearly double, increasing metabolizable energy by 27%. These developments are a result of microbial protein synthesis, enzymatic breakdown of insoluble fiber and the release of bound lipids from the fiber matrix (Fakas 2017; Wang et al. 2024). All of these developments make RB an extremely nutrient‐dense feedstuff, especially in non‐ruminant nutrition where digestibility is a major limitation.

Rice polish showed well‐balanced nutritional changes, such as 18.2% reduction in fiber, 66% increase in ether extract and an 8.8% rise in energy values. While protein changes were minimal, rises in lipid and energy fractions make RP an efficient supplement to animal diets (Khalique et al. 2003). However, DORB exhibited the largest ether extract rise, in addition to significant improvement in protein and loss in fiber. These findings show that fermentation counteracts the initial poor fermentability of the substrate by enhancing its nutritional value to the extent that it is usable as a low‐cost feed ingredient even with low ethanol potential (Khalique et al. 2004).

The similar reduction of nitrogen‐free extract (NFE) for all substrates is indicative of microbial consumption of soluble carbohydrate, and the increase in ash content is due to concentration due to fiber degradation. Combined, these changes agree with previous results confirming fermentation as a good process for the improvement in quality and digestibility of nutrients from agro‐industrial by‐products (Terefe et al. 2021).

### 4.5 Phosphorus Bioavailability

The greatest observation recorded was the dramatic increase in phosphorus availability, namely the reduction of phytate phosphorus. Phosphorus available increased by 55.6% in RB, and the same improving trend was observed in RP and DORB. This is as expected due to phytase‐catalyzed hydrolysis of phytates under fermentation, as revealed by Jatuwong et al. (2020). The same findings have been observed in earlier studies, where phytate levels were reduced considerably by solid‐state fermentation with fungi or yeast and augmented release of phosphorus (Selle and Ravindran 2008; Bingöl et al. 2009). The increments are of particular importance to non‐ruminant animals, to which phytate phosphorus is most unavailable, and inorganic phosphorus supplementation is costly (Lei et al. 2013; Bedford and Cowieson 2012). Fermentation thus possesses the twin advantage of increasing nutritional value along with reducing dependence on expensive inorganic phosphorus additives.

This reduction in PP:AvP ratio in RB, RP, and DORB always indicates increased mineral availability. The optimum recoveries occurred at 30–36 h, which suggests that this time provides the most acceptable balance between microbial metabolism, addition of nutrients, and mineral liberation. Such a result is consistent with earlier findings whereby yeast fermentation efficiently enhanced mineral solubility and bioavailability in cereal by‐products (Selle and Ravindran 2008; Woyengo and Nyachoti 2013). Such a result upholds yeast fermentation not only as a tool for energy and protein enhancement but as an environmentally sustainable tool for maximizing mineral efficiency in animal nutrition (Selle et al. 2000; Ravindran et al. 2014).

Generally, yeast fermentation improved the nutritional value and phosphorus bioavailability of rice milling by‐products including rice bran (RB), rice polish (RP), and de‐oiled rice bran (DORB). RB showed the best fermentation activity, while RP exhibited constant but reduced fermentation, and DORB showed the lowest yield. The three substrates showed improved nutrient composition and increased phosphorus availability. Moreover, several findings revealed that using these by‐products with several enzyme or supplement in different diet can improve performance (Ranjan, Sahu, Deo, Kumar, Kumar, and Jain 2018; Ranjan et al. 2017; Kumar et al. 2017), where fermentation adds those benefits and can effectively be used in animal diet with better growth (Ahmad et al. 2019; Alauddin et al. 2016; Haryati et al. 2015). For further analysis, in vivo trial should perform to establish those criteria as efficient yeast condition as well as making pathway to collect ethanol and used as energy source.

## 5 Conclusion

In general, yeast fermentation significantly added value to rice mill by‐products. RB fermented best and gave the highest yield of ethanol; RP gave moderate yet regular fermentation, while DORB, which gave low gas and ethanol production, still increased nutritive value. Fermentation enhanced crude protein, ether extract, metabolizable energy, and available phosphorus in all the mentioned substrates and reduced crude fiber and phytate. Finally, these findings confirm that the solid‐state yeast fermentation (2.0% yeast, 60% moisture, 30–36 h) is a realistic option to enhance feed quality and mineral availability with further scope for bioethanol production.

## Author Contributions

**Syful Islam:** conceptualization (equal), data curation (supporting), formal analysis (equal), methodology (equal), visualization (equal), writing – original draft (equal). **Md. Abdullah Bijoy:** data curation (equal), investigation (equal), methodology (equal), validation (equal), writing – original draft (equal). **Md. Aliar Rahman:** formal analysis (equal), investigation (equal), software (equal), supervision (equal), writing – review and editing (equal). **Rakhi Chowdhury:** resources (equal), supervision (equal), validation (equal), writing – review and editing (lead). **Khan Md. Shaiful Islam:** conceptualization (equal), funding acquisition (equal), methodology (equal), project administration (equal), supervision (lead), writing – review and editing (lead).

## Ethics Statement

The authors have nothing to report.

## Conflicts of Interest

The authors declare no conflicts of interest.

## Data Availability

The data that support the findings of this study are available from the corresponding author upon reasonable request.
